# A whiff of the future: functions of phenylalanine‐derived aroma compounds and advances in their industrial production

**DOI:** 10.1111/pbi.13863

**Published:** 2022-06-30

**Authors:** Oded Skaliter, Yarin Livneh, Shani Agron, Sharoni Shafir, Alexander Vainstein

**Affiliations:** ^1^ Institute of Plant Sciences and Genetics in Agriculture, The Robert H. Smith Faculty of Agriculture, Food and Environment The Hebrew University of Jerusalem Rehovot Israel; ^2^ Department of Neurobiology The Weizmann Institute of Science Rehovot Israel; ^3^ B. Triwaks Bee Research Center, Department of Entomology, Institute of Environmental Sciences, Robert H. Smith Faculty of Agriculture, Food and Environment The Hebrew University of Jerusalem Rehovot Israel

**Keywords:** aroma compounds, heterologous systems, metabolic engineering, microbes, phenylalanine, phenylpropanoids

## Abstract

Plants produce myriad aroma compounds—odorous molecules that are key factors in countless aspects of the plant's life cycle, including pollinator attraction and communication within and between plants. For humans, aroma compounds convey accurate information on food type, and are vital for assessing the environment. The phenylpropanoid pathway is the origin of notable aroma compounds, such as raspberry ketone and vanillin. In the last decade, great strides have been made in elucidating this pathway with the identification of numerous aroma‐related biosynthetic enzymes and factors regulating metabolic shunts. These scientific achievements, together with public acknowledgment of aroma compounds' medicinal benefits and growing consumer demand for natural products, are driving the development of novel biological sources for wide‐scale, eco‐friendly, and inexpensive production. Microbes and plants that are readily amenable to metabolic engineering are garnering attention as suitable platforms for achieving this goal. In this review, we discuss the importance of aroma compounds from the perspectives of humans, pollinators and plant–plant interactions. Focusing on vanillin and raspberry ketone, which are of high interest to the industry, we present key knowledge on the biosynthesis and regulation of phenylalanine‐derived aroma compounds, describe advances in the adoption of microbes and plants as platforms for their production, and propose routes for improvement.

## Introduction

Aroma compounds are volatile molecules produced by plants, microbes, and animals that are detected by the olfactory system and therefore have an odour (smell) (Schwab *et al*., 2008). Volatile molecules are characterized by low molecular weight, high vapour pressure at ambient temperature and lipophilicity—physical properties that enable them to cross biological barriers and be carried by air to olfactory systems (Pichersky *et al*., 2006; Rowe and Shepherd, 2016). The word ‘aroma’ originates from the Greek word *arōmat* which means ‘spice’, and is defined as ‘a distinctive, pervasive, and usually pleasant or savory smell’ in the Merriam‐Webster dictionary. As sessile organisms, plants use aroma compounds as a means of overcoming the limitations of a stationary existence. Their significance is manifested in their involvement in countless processes throughout the plant's life cycle: from adaption to changing environments and defence against herbivores and pathogens, to plant–plant, plant–microbiome, and plant–pollinator interactions (Ameye *et al*., 2018; Kong *et al*., 2020; Muhlemann *et al*., 2014; Ninkovic *et al*., 2019). To date, myriad aroma compounds have been identified and can be classified as terpenoids, phenylalanine (Phe) derivatives, fatty acid derivatives, and nitrogen/sulphur‐containing compounds (Knudsen *et al*., 2006). Phe‐derived aroma compounds are highly abundant in plants, and many of the aroma compounds desired by man, such as eugenol (clove), raspberry ketone (RK) (raspberry), and vanillin (vanilla) belong to this class (Table 1). Due to their cardinal importance to plants, animals, and humans alike, the *in planta* biosynthesis of Phe‐derived aroma compounds has been extensively studied in a wide range of species, and numerous genes and transcription factors involved in their production and emission have been functionally characterized (Akhtar and Pichersky, 2013; Farhi *et al*., 2010; Klempien *et al*., 2012; Liao *et al*., 2020a; Liu *et al*., 2017; Mageroy *et al*., 2012; Orlova *et al*., 2006; Spitzer‐Rimon *et al*., 2010, 2012). Today, the production of aroma compounds is a multibillion dollar industry, estimated at more than 5 billion USD (Ahuja and Sonal, 2020). Although some of these may be chemically mass‐produced, there is growing consumer demand for biologically produced aroma compounds. Nevertheless, host plants are usually not good sources for the mass production of aroma compounds as they are found in minute quantities, are highly affected by environmental changes, and require laborious extraction processes.

**Table 1:** List of chemical structures, aromas, and plant hosts of Phe‐derived aroma compounds

Compound	Chemical structure	Aroma	Plants
C6–C1 benzenoids			
Benzaldehyde	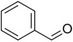	Almond	*Prunus amygdalus*
Benzyl acetate	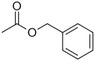	Fresh, jasmine and pear‐like	*Jasminum*
Benzyl alcohol	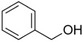	Mild aromatic	*Jasminum*
Benzyl benzoate	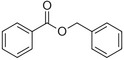	Light, balsamic, reminiscent of almond	*Myroxylon balsamum*
Cinnamaldehyde	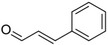	Characteristic cinnamon, pungent‐spicy	*Cinnamomum verum*
Cinnamyl alcohol	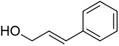	Warm‐balsamic, floral, sweet	*Cinnamomum verum, Myroxylon balsamum*
Guaiacol	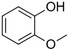	Smokey	*Guaiacum*
Methyl benzoate	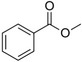	Strong sweet‐floral with a fruity undertone	*Antirrhinum majus*, *Petunia*
Methyl salicylate	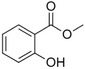	Sweet wintergreen mint, reminiscent of root beer	*Betula lenta, Gauitheria procumbens*
Veratrole	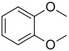	Creamy‐sweet	*Silene latifolia*
C6–C2 phenylpropanoid‐related			
Acetophenone	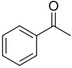	Resembling oranges	*Camellia sinensis*
Phenylacetaldehyde	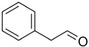	Green floral, rose‐like	*Rosa*
Phenylacetonitrile	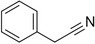	Aromatic	Brassicales
1‐Phenylethanol	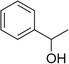	Mild flowery, gardenia–hyacinth	*Camellia sinensis*
2‐Phenylethanol	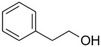	Mild rose‐like, honey	*Rosa*
Phenylethyl acetate	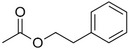	Rose	*Rosa*
Phenylethyl benzoate	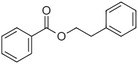	Rose, balsamic	*Cinnamomum zeylanicum*
C6–C3 phenylpropanoids			
Anethole	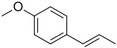	Anise	*Pimpinella anisum*
Chavicol	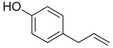	Herbal	*Ocimum basilicum*
Estragole	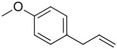	Sweet, reminiscent of anise	*Ocimum basilicum, Malus domestica*
Eugenol	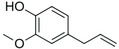	Clove	*Syzygium aromaticum*
Isoeugenol	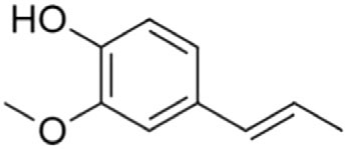	Clove	*Syzygium aromaticum*
Methyl eugenol	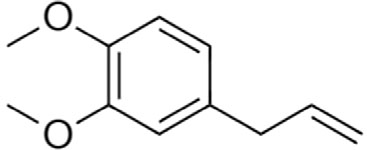	Mild‐spicy, carnation	*Croton malambo*
Methyl isoeugenol	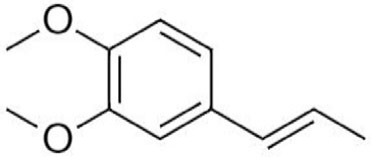	Delicate clove–carnation	*Daucus carota*
Vanillin	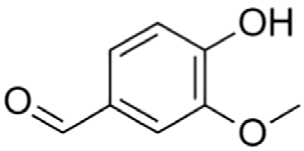	Vanilla, creamy	*Vanilla planifolia*
C6–C4 phenylbutanones			
Raspberry ketone	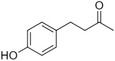	raspberry	*Rubus idaeus*
Zingerone	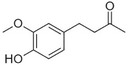	Pungent odour, ginger	*Zingiber officinale*

Owing to their economic, agricultural, and industrial value, enhancement of the production of specialized metabolites in general, and aroma compounds in particular, by host plants and in heterologous systems has garnered massive attention in the last decade, manifested by a plethora of research papers and reviews tackling this topic. The goal of the present review is to give a broader perspective on plant Phe‐derived aroma compounds, from their interactions with other organisms to their production in plants and heterologous systems. The first part of the review presents plant aroma compounds' importance for humans, pollinators, and plant–plant interactions. The second part focuses on the biosynthesis of Phe‐derived aroma compounds and regulation of their fluxes in plants. The final section discusses aroma compound production in plants and heterologous systems, focusing on the most popular Phe‐derived compounds for consumers, RK, and vanillin.

## Aroma compounds' interactions with organisms

### Aroma and humans

**Figure 1:**
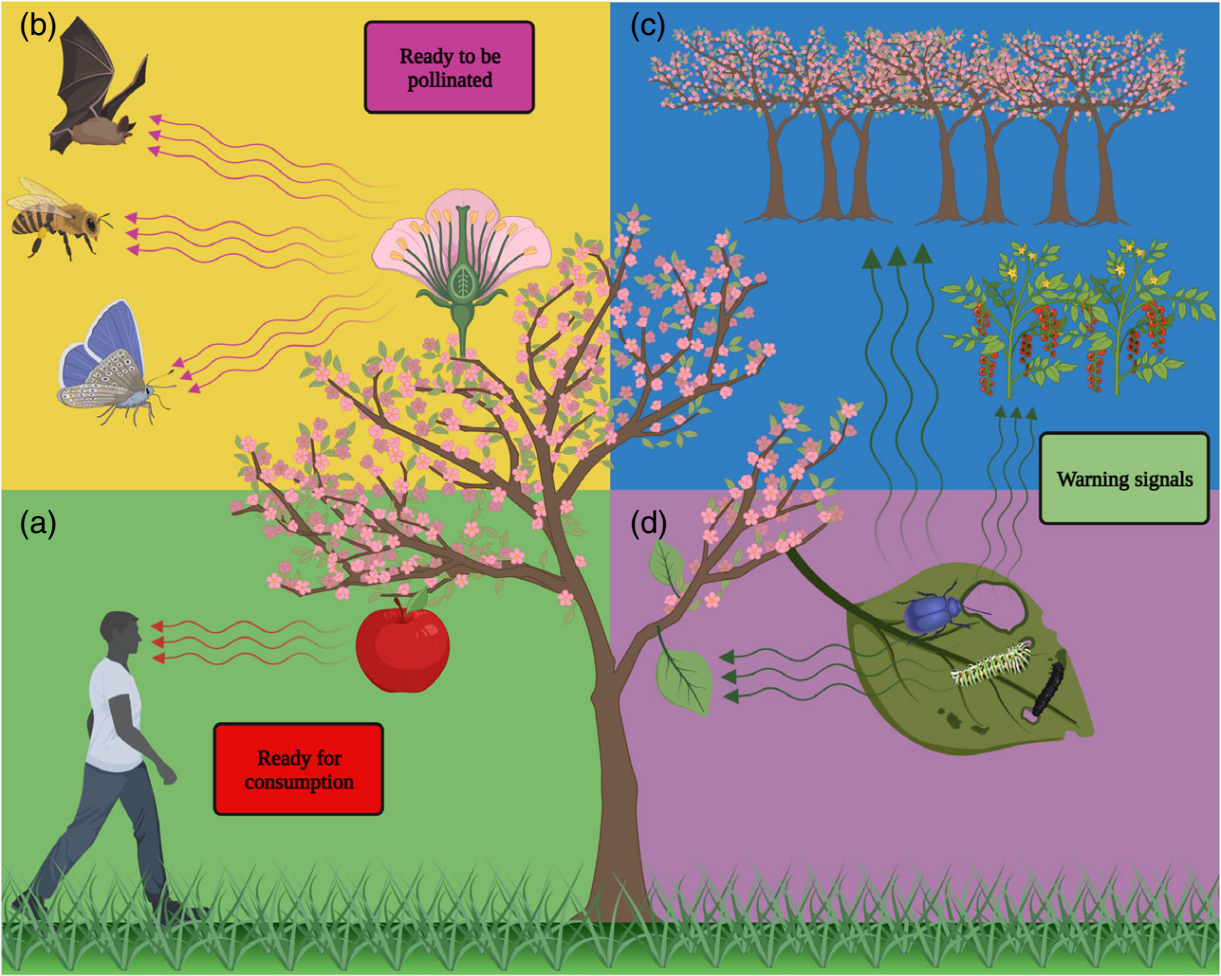
Schematic presentation of interactions between plant‐derived aroma compounds and humans and pollinators, and within and between plants. (a) Ripe fruit releases aroma compounds perceived by the olfactory system. (b) Flowers that are ready for pollination release aroma compounds that make them more conspicuous, attracting, and guiding potential pollinators. (c–d) herbivore infestation may elicit the release of airborne signalling volatiles from damaged tissue that act *in planta* (c) or warn neighbouring plants (d).

Aroma compounds are involved in our everyday lives. Smelling allows us to evaluate our surroundings and is used for hazard avoidance, reproduction, and social interactions. Food is a major aspect of the human–volatile relationship as aroma compounds convey the most accurate information on a food's availability and quality (Fig. 1a). Perhaps, the most important aspect is the contribution of aroma compounds to foods' flavour, and it is no wonder humans have been using them for this purpose since ancient times (Schwab *et al*., 2008). For example, residues of vanillin were identified in ceramic juglets in Tel Megiddo, Israel, dating back ca. 3500 years (Linares *et al*., 2019). The connection between food and aroma goes even deeper as aroma compounds can shape humans' food preference and even influence their eating behaviour (Rolls *et al*., 1996; Small and Green, 2011). For example, aroma intensity of vanilla in a custard dessert affected food intake, with higher intensity resulting in smaller bite size (de Wijk *et al*., 2012). In addition, aroma compounds are suggested to possess health benefits; Phe‐derived compounds like vanillin and cinnamaldehyde have demonstrated anticarcinogenic properties, and RK aids in weight loss (Ahn *et al*., 2015; Ayseli and Ipek Ayseli, 2016; King *et al*., 2007; Morimoto *et al*., 2005). Due to their immense contribution, Phe‐derived compounds, for example, eugenol (clove), cinnamaldehyde (cinnamon), phenylacetaldehyde (rose), benzaldehyde (almond), RK (raspberry) and of course vanillin (vanilla), are highly desired in the food and beverage and pharma industries. The specific rise in demand for ‘natural’ aroma compounds of biological origin has led several large food companies to pledge complete removal of artificial flavours from their products (Bomgardner, 2016). This has ignited extensive research into finding platforms for the production of these compounds (see Microbes and plants as platforms for aroma compound production). In addition, in crops that are consumed fresh, such as tomato, basil, and strawberry (Fan *et al*., 2021; Tieman *et al*., 2017; Walters *et al*., 2021), much effort is being made to identify flavour‐contributing Phe‐derived aroma compounds and the levels that appeal to consumers. Integrating knowledge on the interplay between humans and aroma molecules may enable generating healthier and tastier foods and crops to improve humans' well‐being.

### Aroma and pollinators

As sessile organisms, plants are dependent on external factors for sexual reproduction; ca. 87.5% of angiosperms are pollinated by animals, including birds, mammals and reptiles, with insects being the dominant taxa (Ollerton, 2017). Flowers advertise themselves to pollinators by various cues, olfactory ones playing a major role (Fig. 1b; Joffard *et al*., 2020). Phe‐derived volatile compounds are widespread among floral blends, and benzaldehyde, methyl salicylate (MeSa), benzyl alcohol, and 2‐phenylethanol are the most common (Knudsen *et al*., 2006). These aroma compounds strongly contribute to specificity in plant–pollinator interactions. For example, loss of benzaldehyde emission in *Capsella* underlies the transition from animal‐mediated pollination to selfing (Jantzen *et al*., 2019; Sas *et al*., 2016). In moth‐pollinated petunia, loss of benzaldehyde production led to a shift in pollinator to hummingbirds, which depend largely on their eyesight (Amrad *et al*., 2016). Directed evolution by bumble bee pollination of *Brassica rapa* led to increased floral emission of benzyl nitrile (Ramos and Schiestl, 2019), another Phe‐derived volatile (Liao *et al*., 2020b). From a human perspective, animal pollination is especially important because it is responsible for 35% (by volume) of crop production (Klein *et al*., 2007). Perils such as global environmental changes present a tangible threat to volatile‐mediated plant–pollinator mutualism (Byers and Chang, 2017; Glenny *et al*., 2018). A standing question is whether advanced breeding techniques can be harnessed to improve (or at least maintain) pollination efficiencies to overcome future problems (Dudareva *et al*., 2013). Success has been extremely limited so far, as numerous complexities still need to be addressed. For example, floral bouquets are typically comprised of complex blends of numerous volatiles originating from various biochemical pathways. Increasing the metabolic flux into multiple distinct branches belonging to often competing pathways is extremely complicated, especially when they are negatively correlated, compete for the same substrates, or affect other agriculturally important traits (Iijima *et al*., 2004). Furthermore, pollinators often perceive blends as separate from the components that make up the blend (Wycke *et al*., 2020). Understanding pollinator‐specific perceptual characteristics of components and blends is especially important when planning which compound/s to target. Solving these pending problems is a prerequisite for success in this endeavour.

### Aroma as defence signals within and between plants

In addition to pollination, plants also make use of volatile compounds as part of their defence mechanism, and they are used in both intra‐ and inter‐plant communication (Fig. 1c,d) (Ninkovic *et al*., 2020; Vlot *et al*., 2020). Phe‐derived aroma compounds, such as eugenol, vanillin and guaiacol, have been shown to possess antimicrobial properties (Fitzgerald *et al*., 2004; Friedman *et al*., 2002; Walsh *et al*., 2019; Wang and Fan, 2014). The volatile methyl ester of salicylic acid (SA), MeSA, is a signalling phytohormone that upon conversion to bioactive SA, regulates or activates resistance mechanisms (Park *et al*., 2007; Shulaev *et al*., 1997; Vlot *et al*., 2009). MeSA is a mobile signal that has been shown to travel through the phloem from infected to uninfected tissues, enabling activation of systemic acquired resistance (Park *et al*., 2007). In addition, MeSA is suggested to act as an airborne signal between neighbouring plants; MeSa produced in response to viral infection of tobacco plants activated defence mechanisms in its healthy, uninfected neighbours (Shulaev *et al*., 1997). Although some possible pathways have been suggested, the exact mechanisms of volatile signal perception in plants remain to be investigated (Ninkovic *et al*., 2020). Furthermore, MeSA has been shown to serve indirectly against herbivores by recruiting their natural predators (Ament *et al*., 2010; Rowen *et al*., 2017).

Generating plants with modified emission levels of MeSA and other defence volatiles to improve the defence efficiency and priming of plant populations against pathogens and herbivores seems like a logical goal. Nevertheless, tampering with the defence mechanism might be a double‐edged sword because predicting all layers of volatile‐mediated plant–plant and plant–pathogen interactions is extremely complex. For instance, synthetic lures containing MeSA placed in tomato fields improved resistance to both pathogens and herbivores, but increased their susceptibility to colonization by thrips (Rowen *et al*., 2017). Taken together, although the ability to generate tailor‐made metabolically engineered plants with superior volatile‐based defence mechanisms or alert systems is an attainable goal, a better understanding of the many aspects of plant volatiles' interactions with the environment is required.

Thus, future goals of harnessing Phe‐derived aroma compounds for human needs are not an easy task, because these volatiles are entangled in many of the plant's interactions with the environment that are vital to humans, pollinators, and plants. Hence, the engineering approach to reach these goals needs to be more ‘holistic’ and less ‘atomistic’, due the many layers of aroma function. For instance, MeSA and guaiacol are disliked by consumers and in tomatoes, reducing their levels has been suggested for flavour improvement (Tieman *et al*., 2017). Yet, lowering their levels might compromise the plants and their population defence and priming mechanisms, rendering the plants more susceptible to pathogens and herbivores. MeSA is also a prevalent compound in floral bouquets with a proven role in pollinator attraction (Hoballah *et al*., 2005; Knudsen *et al*., 2006), hence perturbing its synthesis might also have adverse effects on pollination efficiency. Taken together, it is clear that future strategies for metabolic engineering of aroma compounds in plants should ideally consider integration of flavour, pathogen and herbivore resistance, pollination efficiency and more.

## Phe‐derived aroma compound production and regulation in plants

### Phenylpropanoid pathway

**Figure 2:**
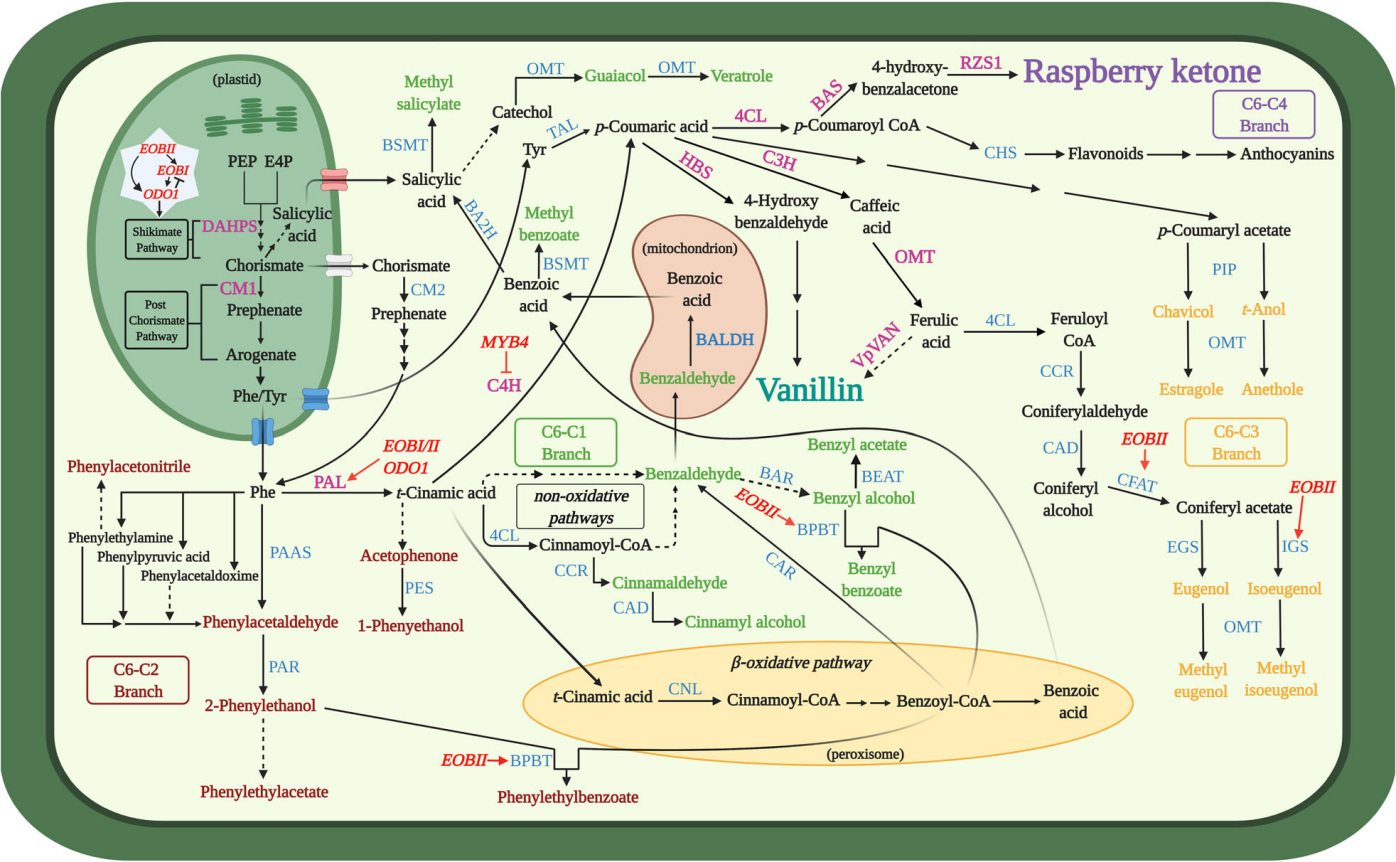
Schematic presentation of biosynthetic pathways leading to the Phe‐derived aroma compounds in plants. L‐phenylalanine (Phe) is synthesized in the plastid or in the cytosol from chorismate generated via the shikimate pathway. Key enzymes in vanillin and raspberry ketone biosynthesis are in magenta. Enzymes involved in the production of other Phe‐derived aroma compounds are in blue, and transcription factors are in red. Dark red, green, orange, and purple indicate aroma compounds originating from the phenylpropanoid‐related (C6–C2), benzenoid (C6–C1), phenylpropene (C6–C3), and phenylbutanone (C6–C4) branches, respectively. Solid arrows indicate established biochemical steps, and dashed arrows indicate hypothetical steps. Stacked arrows represent multiple enzymatic steps. Abbreviations: BAR, benzaldehyde reductase; BAS, benzalacetone synthase; BA2H, benzoic acid 2‐hydroxylase; BALDH, benzaldehyde dehydrogenase; BEAT, acetyl‐CoA:benzylalcohol acetyltransferase; BPBT, benzoyl‐CoA:benzylalcohol/2‐phenylethanol benzoyltransferase; BSMT, benzoic acid/salicylic acid carboxyl methyltransferase; C3H, p‐coumarate 3‐hydroxylase; C4H, cinnamate 4‐hydroxylase; CAD, cinnamyl alcohol dehydrogenase; CAR, carboxylic acid reductase; CCR, cinnamyl‐CoA reductase; CFAT, coniferyl alcohol acetyltransferase; 4CL, 4‐ coumarate‐CoA ligase; CM 1/2, chorismate mutase 1/2; CNL, cinnamoyl‐CoA ligase; DAHPS, 3‐deoxy‐D‐arabino‐heptulosonate 7‐phosphate synthase; E4P, erythrose 4‐phosphate; EGS, eugenol synthase; HBS, hydroxybenzaldehyde synthase; IGS, isoeugenol synthase; OMT, *O*‐methyltransferase; PAR, phenylacetaldehyde reductase; PAAS, phenylacetaldehyde synthase; PAL, phenylalanine ammonia‐lyase; PEP, phosphoenolpyruvate; PES, 1‐phenylethanol synthase, PIP, pinoresinol−lariciresinol reductase, isoflavone reductase, phenylcoumaran benzylic ether reductase; RZS1, raspberry ketone/zingerone synthase 1; TAL, tyrosine ammonia‐lyase; Tyr, tyrosine; VpVAN, vanillin synthase; transcription factors: EOBI/II, emission of benzenoids I/II; MYB4, MYB transcription factor 4; ODO1, odorant 1.

The phenylpropanoid pathway produces myriad metabolites involved in numerous processes of utmost importance in the plant's life cycle, such as lignin, flavonoids, and aroma compounds (Table 1; Vogt, 2010). These metabolites are produced via an intricate and strongly interlinked network of enzymes and regulators. The precursors of the pathway are the aromatic amino acids (AAAs). Biosynthesis of AAAs begins in the plastid‐localized shikimate pathway. In the first step, the enzyme 3‐deoxy‐D‐arabino‐heptulosonate‐7‐phosphate (DAHPS) condenses the pentose‐phosphate‐pathway‐derived erythrose‐4‐phosphate (E4P) and glycolysis‐derived phosphoenolpyruvate (PEP) (Fig. 2). Further enzymatic steps lead to the formation of the pathway's final product—chorismate. Chorismate mutase 1 (CM1) subsequently converts chorismate to prephenate, from which Phe may be biosynthesized in plants mainly via the arogenate pathway (Lynch and Dudareva, 2020). However, Phe biosynthesis is not limited to the plastids; as in microbes, it may also occur in the cytosol via the phenylpyruvate pathway with the aid of chorismate mutase 2 (CM2) (Qian *et al*., 2019). Phenylpropanoid production begins with Phe being directed to two branches mediated by competing enzymes, yielding four aroma compound classes based on their carbon backbone: benzenoids (C6–C1), phenylpropanoid‐related compounds (C6–C2), phenylpropenes (C6–C3), and phenylbutanones (C6–C4) (Fig. 2). Metabolic flow towards benzenoids, phenylpropenes, and phenylbutanones is mediated by the action of L‐phenylalanine ammonia‐lyase (PAL), which in the committed step deaminates Phe to *trans*‐cinnamic acid (CA). This constitutes a main transition point of carbon from primary (amino acid) metabolism to phenylpropanoid‐specialized metabolism. Benzenoids production occurs via the peroxisome‐localized β‐oxidative and/or cytosolic non‐β‐oxidative pathways (Widhalm and Dudareva, 2015). CA conversion to *p*‐coumaric acid by cinnamate 4‐hydroxylase (C4H) followed by multiple enzymes, including CoA ligases like 4‐coumarate‐CoA ligase (4CL), directs metabolism toward phenylpropenes, phenylbutanones, lignins, and flavonoids. Recently, a bifunctional Phe/L‐tyrosine ammonia‐lyase (PTAL), which also converts tyrosine to *p*‐coumaric acid, was identified in *Brachypodium distachyon* (Barros *et al*., 2016; Barros and Dixon, 2020). In addition to route through PAL, Phe may be channelled towards the production of C6–C2 phenylpropanoid‐related aroma compounds via different pathways (Fig. 2), as revealed in tomato, petunia, rose and poplar (Farhi *et al*., 2010; Günther *et al*., 2019; Hirata *et al*., 2016; Kaminaga *et al*., 2006; Tieman *et al*., 2006).

### Vanillin and raspberry ketone biosynthesis

Biosynthesis of the phenylbutanone RK [4‐(4‐hydroxyphenyl)butan‐2‐one], which defines the aroma of ripe raspberry fruit, has been fully elucidated (Fig. 2) (Abe *et al*., 2001; Koeduka *et al*., 2011). CA is converted to *p*‐coumaric acid by C4H, a step also shared with vanillin production, followed by the action of 4CL to yield *p*‐coumaroyl‐CoA. In the penultimate step, benzalacetone synthase (BAS) condenses *p*‐coumaroyl‐CoA and malonyl‐CoA, generating 4‐hydroxybenzalacetone. Reduction of 4‐hydroxybenzalacetone to RK is catalysed by raspberry ketone/zingerone synthase 1 (RZS1).

Formation of vanillin (4‐hydroxy‐3‐methoxybenzaldehyde), the main aroma compound in cured extract of *Vanilla planifolia* pods (Gallage and Møller, 2018), has been suggested to occur via several possible routes. Similar to C6–C3 compounds, *p*‐coumaric acid is the precursor for vanillin (Fig. 2) (Kundu, 2017). Chemically, vanillin is a phenolic aldehyde belonging to the C6–C1 group because it undergoes C2 side‐chain shortening (Kundu, 2017). This latter reaction is suggested to be catalysed by the vanillin synthase recently isolated from *V. planifolia* (VpVAN), directly from ferulic acid (Gallage *et al*., 2014, 2018; Kundu, 2017). However, VpVAN's ability to directly generate vanillin was questioned by Yang *et al*. (2017), who reported its involvement in vanillin biosynthesis through conversion of *p*‐coumaric acid to *p*‐hydroxybenzaldehyde (Kundu, 2017). The debate continues, with studies by Chee *et al*. (2017) and Gallage *et al*. (2018) further suggesting VpVAN's activity via ferulic acid, although detailed enzymological data, such as kinetics, are lacking. For comprehensive reviews of Phe‐derived volatile biosynthesis, we suggest Vogt *et al*. (2010), Muhlemann *et al*. (2014) and Widhalm *et al*. (2015).

### Regulation of Phe‐derived aroma compound biosynthesis and emission

#### Downstream products

Metabolic fluxes in the shikimate and post‐chorismate pathways, which generate the primary precursors of the phenylpropanoid pathway—the AAAs, are regulated by an intricate network involving phytohormones, transcriptional regulators, and negative and positive feedback by downstream products (Fenske *et al*., 2015; Liu *et al*., 2017; Lynch *et al*., 2020; Lynch and Dudareva, 2020; Spitzer‐Rimon *et al*., 2010, 2012; Tzin and Galili, 2010; Verdonk *et al*., 2005). DAHPS is the key enzyme controlling carbon flux into the shikimate pathway (Fig. 2), and therefore a major target for metabolic engineering in both microbes and plants (see Microbes and plants as platforms for aroma compound production). Although in bacteria, DAHPS is allosterically inhibited by the AAA product (Luttik *et al*., 2008; Tzin *et al*., 2012; Tzin and Galili, 2010), until recently, the data for plants were generally lacking (Liao *et al*., 2020a; Yokoyama *et al*., 2021). Yokoyama *et al*. (2021) demonstrated that in *Arabidopsis*, DAHPS is feedback‐inhibited by multiple downstream products, that is, tyrosine, tryptophan, arogenate, caffeate, and chorismate. Another possible limiting factor is availability of E4P for AAA synthesis, as plant DAHPS has a lower *K*_m_ value for PEP than for E4P (Yokoyama *et al*., 2021). In the post‐chorismate stages of the pathway, chloroplast‐localized CM1 is inhibited by Phe and tyrosine and activated by tryptophan. AAAs also inhibit their corresponding producing enzymes (Lynch and Dudareva, 2020; Tzin and Galili, 2010). In the cytosol, Phe controls the phenylpyruvate pathway by inhibiting conversion of prephenate to phenylpyruvate driven by prephenate dehydratase. The next key enzyme in this stream, which is tightly regulated both transcriptionally and enzymatically, is PAL, which is feedback‐inhibited by multiple downstream products, including flavonoids (Zhang and Liu, 2015).

#### Transcription factors

The identification of transcriptional regulators of volatile phenylpropanoid production has been the focus of many studies performed mainly with *Petunia*, a model plant for the study of Phe‐derived aroma compounds (Fig. 2). These studies revealed that a network of MYB‐family transcription factors regulate the biosynthesis of aroma compounds by directly activating shikimate and post‐chorismate pathways, and PAL and aroma‐related genes (Boersma *et al*., 2022; Colquhoun *et al*., 2011; Spitzer‐Rimon *et al*., 2010, 2012; Van Moerkercke *et al*., 2011; Verdonk *et al*., 2005) (Fig. 2). These include the triad of interacting MYBs—ODORANT 1 (ODO1) and EMISSION OF BENZENOIDS (EOB) I and II (Fig. 2). The MYB triad mechanism has been suggested to be conserved among plants because, for example, in *Arabidopsis* flowers that do not emit benzenoids, orthologs of EOBs and ODO1 regulate the production of phenylpropanoid‐derived metabolites in the pollen cell wall (Battat *et al*., 2019). In addition to these positive regulators, a negative factor, MYB4 that fine‐tunes the flux towards C6–C1 and C6–C2 compounds by inhibiting C4H, was also revealed (Colquhoun *et al*., 2011).

#### Phytohormones

Transcription factors, in turn, are regulated by phytohormones (Colquhoun *et al*., 2010; Liu *et al*., 2017; Patrick *et al*., 2021; Ravid *et al*., 2017; Underwood *et al*., 2005; Wang *et al*., 2018). Transcript levels of *EOBI* and *II* were significantly lower in *DELLA*‐suppressed petunia flowers, with concurrent reduction in aroma compound emission (Ravid *et al*., 2017). Therefore, in early stages of flower development (buds), gibberellins promote anthocyanins while blocking aroma compound production by mediating the degradation of DELLA proteins. Towards flower opening, gibberellin levels decrease, shifting production from anthocyanins to aroma compounds (Patrick *et al*., 2021; Ravid *et al*., 2017). Similarly, during senescence or after pollination, ethylene levels increase and block the production of aroma compounds, mediated by the action of ETHYLENE RESPONSE FACTOR 6, which interacts with EOBI's DNA‐binding domain, thereby preventing it from activating the promoters of aroma‐related target genes such as *ODO1* (Liu *et al*., 2017; Underwood *et al*., 2005; Wang *et al*., 2018). Jasmonates—phytohormones that play a vital role in plant defence—elicit signalling cascades in response to physical damage, which lead to increased production of phenylpropanoids, Phe, and CA, and upregulation of *PAL* (Gundlach *et al*., 1992; Halitschke and Baldwin, 2004; Hanik *et al*., 2010; Shi *et al*., 2015). Recently, crosstalk between cytosolic Phe synthesis and auxin was identified by overexpression of cytosol‐localized CM2 (Lynch *et al*., 2020). Unexpectedly, in the transgenic CM2‐overexpressing lines, levels of both Phe and downstream compounds decreased, whereas that of auxin increased. The latter negatively affected the development of plastids, the main route for AAA synthesis in plants (Lynch *et al*., 2020).

#### Compartmentalization

The biosynthesis of Phe‐derived aroma compounds depends on the flow of metabolites between chloroplast, cytosol, endoplasmic reticulum, peroxisome, vacuole, and mitochondrion (Fig. 2; Achnine *et al*., 2004; Adebesin *et al*., 2018; Cna'ani *et al*., 2017; Widhalm and Dudareva, 2015). Despite our deeper understanding of the mechanisms of Phe‐derived aroma compound biosynthesis and regulation, to date, little is known about their cellular pathway from generation to release into the atmosphere. The vacuole has been suggested to serve as a mid‐station between aroma compound production and emission (Cna'ani *et al*., 2017; Song *et al*., 2018). Compounds, such as benzyl alcohol, vanillin, eugenol, isoeugenol, and 2‐phenyethanol may undergo glycosylation, which increases their water solubility, reduces their volatility and serves as a translocation signal. Following glycosylation, they are compartmentalized and stored in the vacuole. The glycosylation step is suggested to be part of the aroma‐production mechanism as the glycosylated pool of aroma compounds is dynamic, coinciding with increased emission, both developmentally and diurnally (Cna'ani *et al*., 2017). In addition, glycosylation has been suggested to help maintain cell integrity by detoxifying Phe‐derived compounds that might be damaging to the cell when accumulated to high levels (Adebesin *et al*., 2017). Moreover, excess Phe has also been shown to be stored in vacuoles (Lynch *et al*., 2017). Other organelles also have the ability to store aroma compounds, for example, vanillin glucoside which is stored in specialized plastids named phenyloplasts (Brillouet *et al*., 2014; Gallage *et al*., 2018).

#### Regulation of emission

Until recently, it was generally believed that most aroma compounds are released from flowers by passive diffusion. However, calculations of emission rates revealed that with passive diffusion alone, some aroma compounds would accumulate to toxic levels in the cell membranes, suggesting the existence of active mechanisms (Widhalm *et al*., 2015). Indeed, two factors involved in the active emission of aroma compounds have been identified: the MYB PH4 and adenosine triphosphate‐binding cassette subfamily member 1 (ABCG1) (Adebesin *et al*., 2017; Cna'ani *et al*., 2015). The latter has also been shown to dictate the spatial pattern of emission from floral tissues (Skaliter *et al*., 2021). Whereas the exact mechanism by which PH4 affects emission has yet to be identified, the plasma‐membrane‐localized ABCG1 actively exports aroma compounds from the cell (Adebesin *et al*., 2017). After crossing the plasma membrane and the cell wall, aroma compounds encounter another barrier—the cuticle. Liao *et al*. (2020a) demonstrated that the flower cuticle is not only a passive barrier but also functions as a reservoir for compounds with relatively low volatility, such as benzyl benzoate, isoeugenol, 2‐phenylethanol and benzyl alcohol, and is a crucial part of the mechanism interconnecting production and emission. Flowers with reduced cuticle thickness not only emitted less, but also produced less aroma compounds, and carbon‐labelling indicated that Phe biosynthesis was compromised. Furthermore, DAHPS, CM1 and 2, and ODO1 were downregulated in these flowers, suggesting the activation of a feedback mechanism regulating carbon flux into the pathway aimed at preventing overaccumulation of aroma compounds that are toxic to the cell (Liao *et al*., 2020a). These findings could serve to manipulate the production and emission mechanisms towards the generation of better or stronger smelling fruit and flowers.

#### Conclusions

Overall, to ensure that scent is produced and emitted at the right time and in the right place, multiple players, that is, metabolites, organelles, transcription/epigenetic factors, phytohormones, and physical barriers are interlinked to tightly regulate this high‐energy‐demanding process. When examining this elaborate mechanism, it is clear why metabolic engineering of Phe‐derived aroma compounds is no easy task, as reprogramming the carbon flux with so many players is extremely challenging. For example, although an increase in Phe due to suppression of the three petunia PALs might be expected to lead to redirection of carbon flux towards metabolism of C2 compounds, emission of phenylacetaldehyde and 2‐phenylethanol are not altered. Instead, excess Phe is diverted to an inactive pool in the vacuole (Lynch *et al*., 2017). Furthermore, the crosstalk between the primary and specialized metabolisms has not been fully deciphered, for example, the factors that directly ‘turn on’ the master transcription factor of aroma compound biosynthesis, EOBII, at the onset of aroma compound production in flowers at anthesis are unknown, as are the molecular signals that simultaneously deactivate anthocyanin production. Answering these vital questions will enable more accurate metabolic engineering of aroma compounds.

## Microbes and plants as platforms for aroma compound production

The importance of aroma compounds to the food, beverage, perfume and pharma industries makes them a highly desirable commodity. Some of these compounds can be chemically mass‐produced, but they are perceived as unhealthy; chemical synthesis is often not environmentally friendly and not stereospecific, although the cost of synthetic compounds is far lower than that of natural ones (Dubal *et al*., 2008; Gallage and Møller, 2015; Lee *et al*., 2016; Martínez *et al*., 2017; Ni *et al*., 2015). Furthermore, there is a rise in consumer demand for ‘natural’ aroma products of ‘biological’ origin and the US Food and Drug Administration requires labelling products as ‘flavoured’ or ‘artificial’ if the source of the compound is not natural (Bomgardner, 2016). All of this is driving the need for natural aroma compounds. On the other hand, native plants from which aroma compounds can be extracted are usually not a good source for them: for the most part, these plants are not domesticated and/or cultivated, require prolonged growing periods, and are strongly affected by biotic and abiotic factors; moreover, compound accumulation is specific to developmental stage and tissue, only trace amounts of these compounds are produced, and the extraction process is expensive. Although classical breeding approaches for increased production of specialized metabolites have been demonstrated in, for example, *Malus domestica* (apple) (Chagné *et al*., 2013), melon (Tzuri *et al*., 2015), tomato (Karniel *et al*., 2020), and *Artemisia annua* (Czechowski *et al*., 2020), modern crops are not generally bred for specialized metabolites of interest due to limited gene pools, trait complexity, and the difficulty involved in screening breeding populations (Peled‐Zehavi *et al*., 2015). Therefore, extensive research together with molecular techniques have been applied in plants and microbes to generate advanced platforms for the production of specialized metabolites. Some examples for Phe‐derived aroma compounds include benzaldehyde (Kunjapur *et al*., 2014) in *Escherichia coli*, cinnamaldehyde, and cinnamyl alcohol in yeast (Gottardi *et al*., 2017), and eugenol in poplar and strawberry (Hoffmann *et al*., 2011; Lu *et al*., 2017). Most of the effort, however, has been invested in the production of vanillin and RK, which are highly desired by the industry and share similar issues (Lee *et al*., 2016; Milke *et al*., 2020; Muheim and Lerch, 1999; Padel and Foster, 2005; Román *et al*., 2017; Sinha *et al*., 2008), among them: extraction of ‘natural’ and vanillin and RK is expensive and labour‐intensive, and their yields from host plants are low. To produce 1 kg of vanillin, ca. 500 kg of vanilla pods are required, and for RK yields are even lower, with production of 1 kg requiring ca. 500 tons of raspberries (Gallage and Møller, 2015; Lee *et al*., 2016); estimated prices are ca. 4000 USD/kg and ca. 3000 USD/kg for naturally derived vanillin and RK, respectively, whereas prices for synthetic compounds are hundreds of times lower (Lee *et al*., 2016; Singh *et al*., 2015). In the following sections, we discuss the pros and cons of using microbes and plants as platforms for the production of Phe‐derived aroma compounds, focusing on vanillin and RK. We further highlight production difficulties and bottlenecks, and ways of overcoming them.

### Microbes

Microorganisms, most commonly the bacterium *E. coli* and the yeast *Saccharomyces cerevisiae*, have been widely used as heterologous systems for the production of numerous specialized metabolites. Microbes often lack the complete biochemical pathways for biosynthesis of plant aroma compounds. Therefore, the main approach for metabolic engineering relies on heterologous expression of enzymes to complete the necessary chemical steps, complemented with mutation or deletion of specific genes to direct metabolite flow towards the target while suppressing competing metabolic shunts and preventing unwanted modifications to the product. Microbes have many advantages for metabolic engineering, such as rapid growth, secretion of metabolites to the media for easy harvest, simple transformation procedures and inexpensive carbon and nitrogen sources (Chemler and Koffas, 2008; Miralpeix *et al*., 2013; Yang *et al*., 2020)). Another major advantage of the production of plant metabolites in microbes is that competing branches are often non‐existent, and there is less chance of feedback inhibition by non‐target downstream products, for example, flavonoids in the case of PAL (see earlier). Moreover, both European and US food legislation classifies microbial‐produced aroma compounds as GRAS (generally recognized as safe) (Food and Drug Administration, 2018; Kallscheuer, 2018).

**Figure 3:**
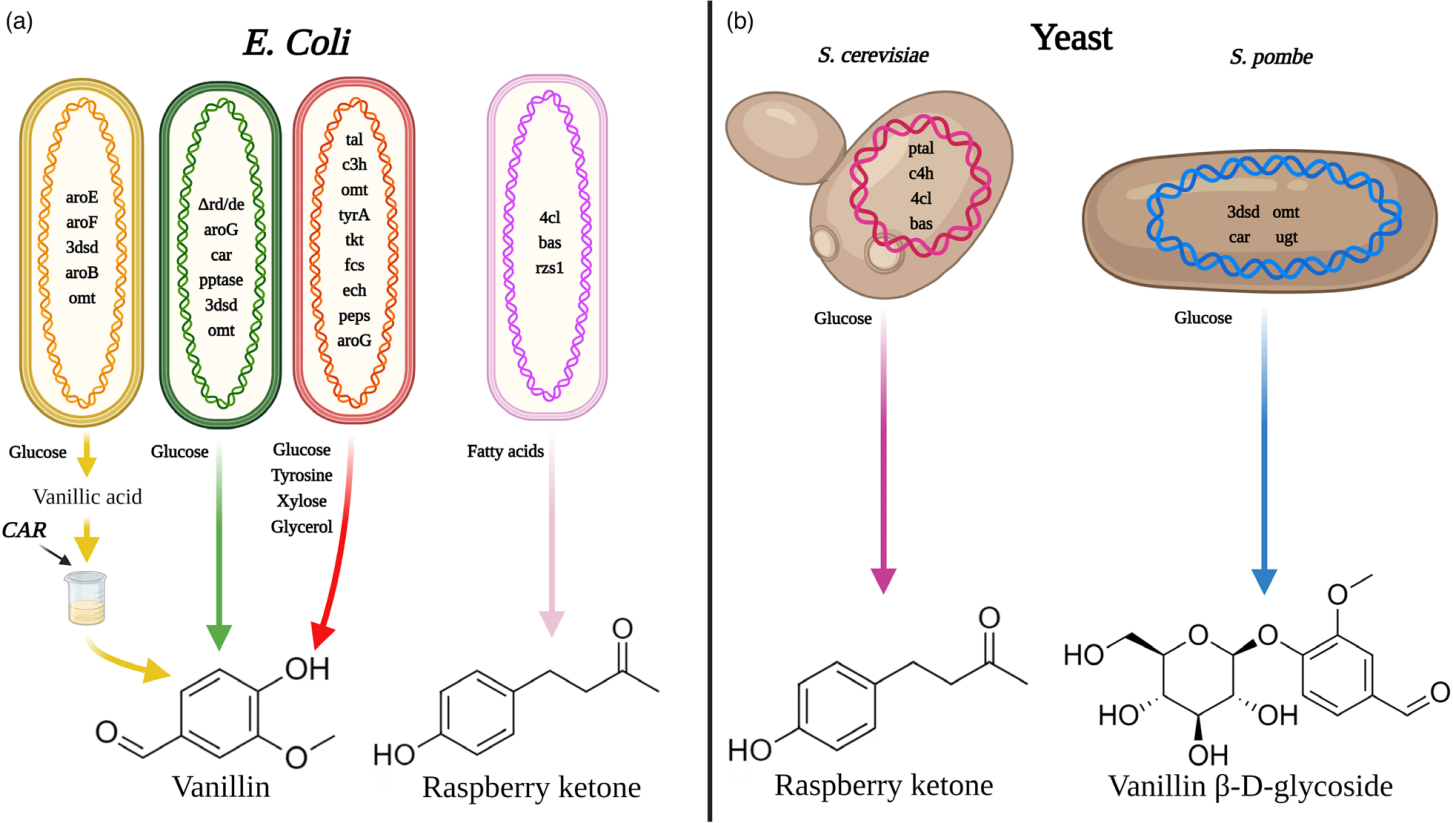
Microbial systems producing vanillin and raspberry ketone. (a) Four metabolically engineered *Escherichia coli* strains from left to right: recombinant strain harbouring a pathway for production of vanillic acid from glucose with subsequent *in vitro* reduction of vanillic acid to vanillin by carboxylic acid reductase (CAR) (Li and Frost, 1998); strain in which genes that encode aldo‐keto reductases and alcohol dehydrogenases (Δrd/de) were deleted together with the introduction of a pathway for the biosynthesis of vanillin from glucose (Kunjapur *et al*., 2014); strain harbouring a pathway mimicking vanillin biosynthesis in plants capable of using L‐tyrosine, glucose, xylose or glycerol as substrates (Ni *et al*., 2015); strain harbouring enzymes for raspberry ketone production from fatty acids (Chang *et al*., 2021). (b) Metabolically engineered *Saccharomyces cerevisiae* and *Schizosaccharomyces pombe* containing the complete pathway for raspberry ketone and vanillin β‐D‐glycoside biosynthesis from glucose (Hansen *et al*., 2009; Lee *et al*., 2016). Abbreviations: 3dsd, 3‐dehydroshikimate dehydratase; aroB, 3‐dehydroquinate synthase; aroE, shikimate dehydrogenase; aroF/G, 3‐deoxy‐D‐arabinoheptulosonate 7‐phosphate synthase; aroZ/asbF, 3‐dehydroshikimate dehydratase; car, carboxylic acid reductase; ech, enoyl‐CoA hydratase/aldolase; fcs, *trans*‐feruloyl‐CoA synthetase; omt, *O*‐methyltransferase; peps, phosphoenolpyruvate synthetase; pptase, phosphopantetheinyl transferase; tkt, transketolase; tyrA, chorismate mutase/prephenate dehydrogenase; tyrR, a regulatory protein; ugt, UDP‐glucuronosyltransferase.

Industrially applicable fungi and bacteria have been successfully metabolically engineered to generate vanillin from different substrates, such as ferulic acid, eugenol and isoeugenol, reaching yields of >10 g/L (Gallage and Møller, 2015). Microalgae can also produce vanillin, albeit at low levels, via biotransformation with ferulic acid (Tripathi *et al*., 2002). Nonetheless, glucose and other carbon sources are considered more ideal substrates as they are readily utilized, non‐toxic, and inexpensive (Gallage and Møller, 2015). Vanillin production from glucose was achieved in *E. coli* harbouring a mutated shikimate dehydrogenase (*aroE*) combined with modifications that allowed bypassing the phenylpropanoid pathway entry points that exist in plants. However, the modified *E. coli* was only able to generate vanillic acid and the reduction to vanillin was performed *in vitro* (Fig. 3a) (Li and Frost, 1998). To eliminate the costly and complicated *in vitro* reaction, yeast with aromatic carboxylic acid reductase (ACAR) and phosphopantetheinyl transferase (PPTase), necessary for ACAR activation, were generated. This created the complete route for vanillin production from glucose, yielding 65 and 45 mg/L vanillin in *Schizosaccharomyces pombe* and *S. cerevisiae* (Fig. 3b), respectively (Hansen *et al*., 2009). The ACAR‐based strategy also proved effective for heterologous production from glucose of the aroma compounds cinnamaldehyde and cinnamyl alcohol (Gottardi *et al*., 2017). However, in this case, the authors ectopically expressed PAL from *Arabidopsis* to generate CA from Phe (instead of dehydroshikimic acid path), enabling *de novo* biosynthesis of the target molecules. Similarly, Ni *et al*. (2015) introduced a synthetic vanillin pathway into *E*. *coli* aimed at mimicking the one in plants, to avoid diversion of substrates from the shikimate pathway. They used tyrosine ammonia‐lyase, which converts tyrosine directly to *p*‐coumaric acid and not PAL, to overcome the low activity of C4H in *E. coli* due to the absence of a suitable NADPH reductase (Fig. 3a) (Watts *et al*., 2004). The constructed pathway allowed the biogenesis of vanillin with media containing tyrosine, reaching a titre of 97.2 mg/L. Furthermore, the modified bacterium could also utilize glucose, xylose, and glycerol (Fig. 3a) (Ni *et al*., 2015).

To construct a pathway for *de novo* biosynthesis of RK in yeast, PTAL, with the ability to use both Phe and tyrosine as substrates, was expressed together with plant C4H, 4CL, and BAS (Fig. 3b) (Lee *et al*., 2016). There was no need to add the enzyme responsible for the final step, benzalacetone reductase (BAR), as the yeast demonstrated endogenous activity (Beekwilder *et al*., 2007). Maximum RK yield was 2.81 mg/L when 4CL and BAS were physically fused, an approach suggested to allow substrate funnelling (Aalbers and Fraaije, 2019; Albertsen *et al*., 2011; Lee *et al*., 2016). Indeed, multiprotein complexes known as ‘metabolons’ have been identified in plants, including PAL and C4H (Achnine *et al*., 2004; Zhang and Fernie, 2021). However, fusion of PTAL and C4H in yeast led to an inverse effect, greatly reducing the final yield of RK (Aalbers and Fraaije, 2019; Lee *et al*., 2016). To our knowledge, *de novo* RK biosynthesis has not been achieved in *E*. *coli*; however, substantial amounts have been obtained by growing *E*. *coli* engineered with 4CL, BAS and raspberry ketone/zingerone synthase 1 (RZS1) on fatty acids, reaching a titre of 180.94 mg/L (Fig. 3a) (Chang *et al*., 2021).

The importance of the shikimate and AAA pathways as the primary source for downstream metabolites has established them as a key target for metabolic engineering (Averesch and Krömer, 2018; Peled‐Zehavi *et al*., 2015). Both pathways are tightly regulated, with significant bottlenecks due to feedback inhibition of two key enzymes—DAHPS and CM—by their products, the AAAs (see Regulation of Phe‐derived aroma compound biosynthesis and emission). Use of feedback‐insensitive forms of these enzymes has proven successful in alleviating these bottlenecks in microbes and plants (Graf and Altenbuchner, 2014; Liu *et al*., 2019; Luttik *et al*., 2008; Oliva *et al*., 2017; Tzin *et al*., 2012, 2013; Xie *et al*., 2016). This approach was also taken for the de novo biosynthesis of vanillin in microbes. Li and Frost (1998), Kunjapur *et al*. (2014) and Ni *et al*. (2015) all introduced a feedback‐insensitive DAHPS into their modified *E. coli* for improved carbon flux. In yeast, strains overexpressing an insensitive DAHPS (Aro4) exhibited a three‐fold increase in AAAs, together with an increase in the Phe‐derived aroma compounds 1‐phenylethanol and phenylacetate (Liu *et al*., 2019; Luttik *et al*., 2008). Combining Aro4 with an insensitive CM (Aro7) further increased flux towards the AAA, manifested by enhanced production of *p*‐coumaric acid, a precursor of both vanillin and RK (Liu *et al*., 2019). E4P of the pentose phosphate pathway is another limiting factor in microbes, because DAHPS has low affinity to it and its endogenous pool is low. To overcome this, Liu *et al*. (2019) expressed two phosphoketolases to divert the carbon flux from glycolysis towards E4P. Indeed, combining phosphoketolases together with Aro4 and 7 further increased the levels of *p*‐coumaric acid (Liu *et al*., 2019).

One of the problems in attempting to enhance metabolite production in microbes is conversion of the target molecule to unwanted side products. More specifically, as microbes rapidly convert aldehydes to alcohols, vanillin and its precursor protocatechualdehyde may be converted to the unwanted by‐products, such as vanillyl alcohol and protocatechuic alcohol respectively. A rational approach to overcoming this problem is to delete or knock out the genes encoding the enzymes with this activity. In this way, Kunjapur *et al*. (2014) generated *E. coli* with 55 times higher titres of vanillin (119 mg/L) compared with *E. coli* without deletion of the respective reductases (Fig. 3a). These strains are also capable of accumulating the benzenoid benzaldehyde, as its reduction to benzyl alcohol was also decreased (Kunjapur *et al*., 2014). Although successful in this case, deletion or silencing of ‘unwanted’ genes is not always an applicable strategy as these may be involved in other pathways that are crucial for the organism's survival. For example, in *S*. *cerevisiae*, an enoyl reductase that is involved in the biosynthesis of fatty acids also converts *p*‐coumaroyl‐CoA (*p*CA) to phloretic acid, resulting in carbon loss when attempting to enhance phenylpropanoid (i.e., flavonoid) production (Lehka *et al*., 2017). To avoid *p*CA conversion while preserving fatty acid production, two alternative approaches were explored: a mutation in the active site of enoyl reductase responsible for phloretic acid production, and incorporation of a plant gene that lacks this activity. The latter approach was more effective in preventing carbon loss (Lehka *et al*., 2017).

One may think that when it comes to enhancement of aroma compounds, the goal is always ‘the more the merrier’. Nevertheless, aroma compounds in general, and vanillin and RK in particular, become toxic to cells at certain threshold concentrations (Beekwilder *et al*., 2007; Fitzgerald *et al*., 2004; Friedman *et al*., 2002; Hansen *et al*., 2009). Vanillin was shown to inhibit growth of *S*. *cerevisiae* cells at concentrations above 500 mg/L (Hansen *et al*., 2009). Similarly, vanillin demonstrated bacteriostatic properties in *E*. *coli* via disruption of the membrane, ultimately impairing respiration (Fitzgerald *et al*., 2004); thus, the rapid reduction of vanillin to the by‐product vanillyl alcohol in that organism is probably a detoxification step (Graf and Altenbuchner, 2014). Interestingly, membrane disruption was also reported in petunia cells in which the Phe‐derived volatile release mechanisms were impaired (Adebesin *et al*., 2017; Liao *et al*., 2020a). Another approach to lowering a compounds' toxicity is by modification, such as methylation or glycosylation. Indeed, in *Vanilla* pods, vanillin is stored as vanillin β‐D‐glucoside, and *S*. *cerevisiae* cells grown on media supplemented with vanillin β‐D‐glucoside at 25 g/L (50‐fold more than vanillin) do not exhibit impaired growth (Hansen *et al*., 2009). To obtain glycosylation of vanillin in yeast, a glycosyltransferase from *Arabidopsis* was incorporated. Indeed, it successfully converted almost all of the vanillin to this less toxic water‐soluble form, yielding 500 mg/L of vanillin β‐D‐glucoside (Fig. 3b) (Brochado *et al*., 2010; Hansen *et al*., 2009). Nonetheless, when the desired product is the aglycone, and not the glycoside, another step is required to break the glycoside residue.

The studies described in this section demonstrate the progress achieved in metabolic engineering of bacteria and yeast for the production of the commercially important aroma compounds vanillin and RK. These advances are due to the improved understanding of metabolic fluxes in host microbes, together with identification of specialized genes from different organisms. Perhaps, the best proof of this progress is the fact that biologically derived vanillin generated from glucose by microbial fermentation is already being sold (Waltz, 2020).

### Plants

With our growing understanding of the metabolic fluxes within and between target pathways, their feedback loops, wide‐scale functional gene analyses, big data analyses, tools for directed evolution of target enzymes, etc., plants are an attractive system for the production of target metabolites. This is further strengthened by the progress that has been made in the application of precise genome‐editing tools (Bortesi and Fischer, 2015; Pott *et al*., 2019; Yang *et al*., 2014). Moreover, plants have some inherent advantages over microorganisms as platforms for the enhancement of endogenous pathways or as hosts for heterologous ones (Owen *et al*., 2017). From an economic standpoint, the production process in plants is autotrophic and more cost‐effective than in microbes, scale up is relatively easy, and there is no need for culture media or special growth facilities (Fu *et al*., 2018). Moreover, as flowers and fruit are, in many cases, the end product, they present an interesting target for metabolic engineering to improve food flavour and nutritional qualities (Ashokkumar *et al*., 2020; Hoffmann *et al*., 2011; Lobato‐Gómez *et al*., 2021; Yu *et al*., 2021).

When dealing with Phe‐derived pathways, plants present the advantage of possessing all of the basic components of the target pathway, as opposed to microbes. Thus, expression of dedicated transcription factors for the activation of multiple genes in a pathway(s) is a routine approach in plant metabolic engineering (Koeduka *et al*., 2021; Skaliter *et al*., 2019; Zhang *et al*., 2015; Zvi *et al*., 2008, 2012). In some cases, the genetic components are not actively producing target products because the substrate is missing, a phenomenon known as ‘silent metabolism’ (Fang *et al*., 2021; Hoffmann *et al*., 2011; Lewinsohn and Gijzen, 2009). For example, lisianthus flowers that are normally unscented produced the Phe‐derived methyl benzoate and 2‐phenylethanol when precursor availability was increased (Fang *et al*., 2021). Another advantage is that the final biosynthetic steps of many specialized metabolites are not fully detailed, and plants offer the possibility to complete these uncharacterized reactions (Dong *et al*., 2012; Jacobowitz and Weng, 2020; Zhou *et al*., 2018). Another main advantage of plants, as eukaryotic organisms, over microbes, is the presence of organelles for example, improved sequestration or accumulation of target molecules, or storage of toxic compounds. On the other hand, until recently, there was no efficient way to achieve targeted plant genome modifications, while growth of transgenic plants and the use of products extracted from them are restricted by complex regulations.

**Figure 4:**
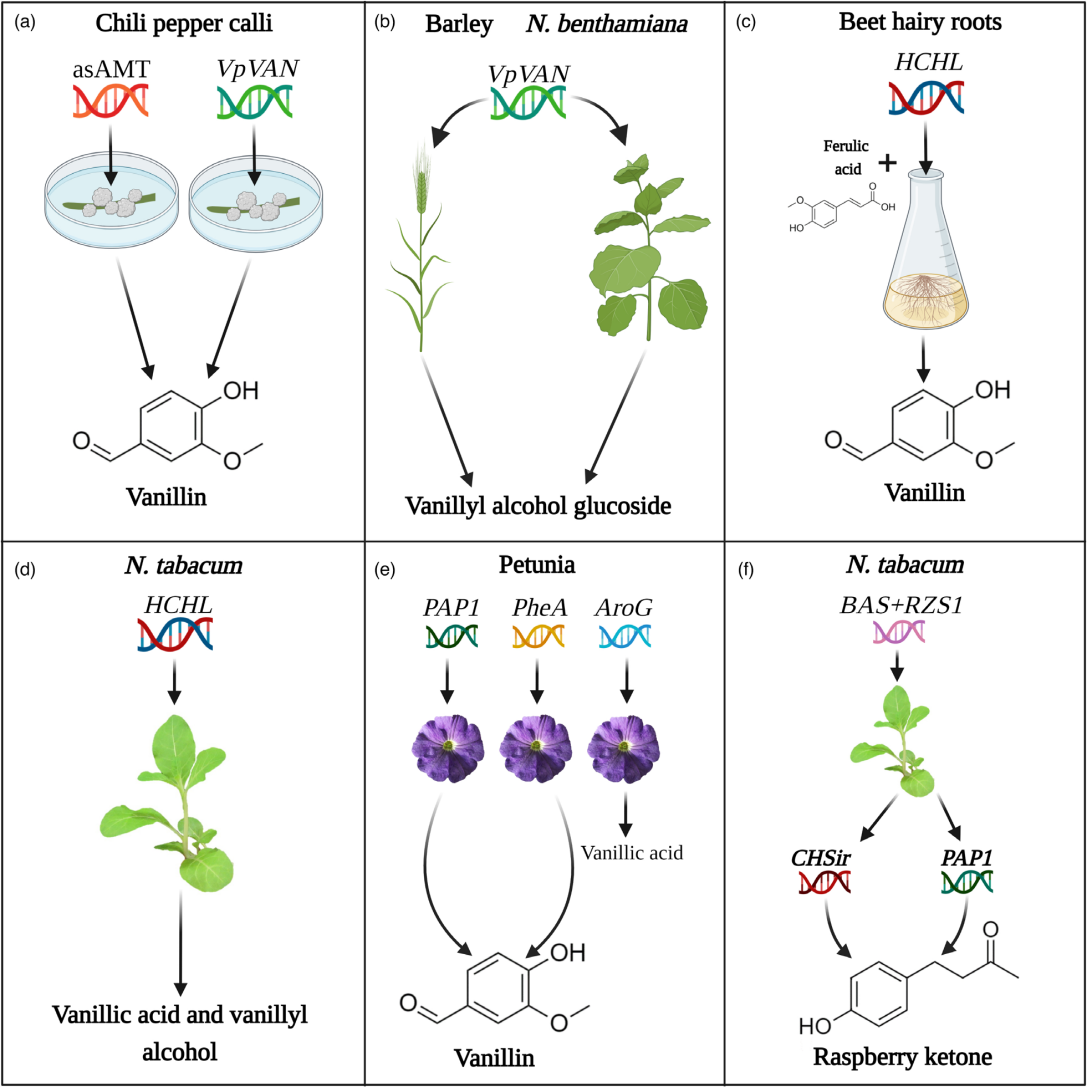
Metabolic engineering of plant‐based systems for enhanced production of raspberry ketone and vanillin. (a, left) Vanillin production in chili pepper (*Capsicum frutescens*) calli following silencing of aminotransferase (AMT) using *Agrobacterium tumefaciens* (agro) carrying an AMT sequence in antisense orientation (asAMT) (Gururaj *et al*., 2012). (a, right and b) *Vanilla planifolia* vanillin synthase (*VpVan*) was introduced into chili pepper calli by particle bombardment (a) and into barley (*Hordeum vulgare*) and *Nicotiana benthamiana* by agro transformation (b) (Chee *et al*., 2017; Gallage *et al*., 2014). (c) *Agrobacterium rhizogenes*‐mediated transformation of beet with 4‐hydroxycinnamoyl‐CoA hydratase/lyase (HCHL) from *Pseudomonas fluorescens*, followed by feeding of transgenic hairy roots with ferulic acid (Singh *et al*., 2015). (d) Ectopic expression of HCHL in transgenic *Nicotiana tabacum* (Mayer *et al*., 2001). (e) Petunia plants genetically engineered to express PRODUCTION OF ANTHOCYANIN PIGMENT 1 (PAP1) from *Arabidopsis thaliana*, bacterial chorismate mutase/prephenate dehydratase enzyme (PheA) or bacterial 3‐deoxy‐D‐arabino‐heptulosonate 7‐phosphate (AroG) (Zvi *et al*., 2008; Cna'ani *et al*., 2014; Oliva et al., 2015, Oliva *et al*., 2017). (f) Ectopic expression of BAS and RZS1 combined with either PAP1 expression or RNAi‐mediated suppression of CHS (CHSir) to generate raspberry ketone in *Nicotiana tabacum* plants (Koeduka *et al*., 2021).

As the pathway/enzymes leading to vanillin production have yet to be fully characterized, hot chili pepper (*Capsicum frutescens*) is an example of a potential target for vanillin production, since vanillin is a precursor of capsaicins (pungency factor in hot pepper) (Chee *et al*., 2017; Gururaj *et al*., 2012). Pepper is highly recalcitrant to transformation, and attempts to produce vanillin in pepper have focused on work with calli. Silencing of the aminotransferase responsible for transamination of vanillin to vanillyl amine (the direct precursor of capsaicin) in pepper calli led to increased levels of the former (Fig. 4a) (Gururaj *et al*., 2012; Weber *et al*., 2014). Ectopic expression of *VpVAN* following particle bombardment of chili pepper callus led to substantially enhanced vanillin production (Fig. 4a); transformed calli generated ca. 573.39 μg/g fresh weight (FW) of vanillin, which translates to a 190‐fold increase compared with non‐trasnformed calli (Fig. 4a). Levels of vanillin β‐D‐glucoside also increased ca.16‐fold (110.32 μg/g tissue), revealing an additional pool that might be tapped in the future. It would be interesting to combine both approaches, that is, aminotransferase silencing and *VpVAN* expression. VpVAN was also tested in *Nicotiana benthamiana* and barley, leading to the accumulation of vanillyl alcohol glucoside (Fig. 4b; Gallage *et al*., 2014).

An alternative approach to vanillin production based on *Pseudomonas fluorescens* 4‐hydroxycinnamoyl‐CoA hydratase/lyase (HCHL), which converts the lignin precursor feruloyl‐CoA to vanillin in two steps, was employed in beet (*Beta vulgaris*) hairy root cultures (Fig. 4c; Gasson *et al*., 1998; Singh *et al*., 2015). Vanillin yield was similar to that in pepper callus, reaching 520 μg/g FW. Feruloyl‐CoA is formed from ferulic acid by 4CL (Barros *et al*., 2019). Supplementation of ferulic acid to hairy root lines enhanced vanillin production to 2090 μg/g FW, reaching the yield of the vanilla orchid and revealing ferulic acid as a major production bottleneck. However, ferulic acid is an expensive substrate, rendering this approach cost‐ineffective (Gallage and Møller, 2015). Ectopic expression of HCHL was also attempted in tobacco, which synthesizes vanillin naturally, albeit in trace amounts (Kundu, 2017; Mayer *et al*., 2001). Transgenic HCHL‐expressing plants accumulated increased amounts of the vanillin derivatives, that is, vanillic acid and vanillyl alcohol (Fig. 4d). However, the alterations in metabolic flux resulted in multiple phenotypic abnormalities, such as phloem fiber deformation. These results demonstrate the negative ramifications of perturbing crucial metabolic pathways (Mayer *et al*., 2001). Vanillin is also a component of scent bouquets of flowers (Knudsen *et al*., 2006; Oliva *et al*., 2015, 2017; Skaliter *et al*., 2021). In petunia flowers, production of vanillin and its derivatives was enhanced by introducing an activator of the phenylpropanoid pathway—the *Arabidopsis* transcription factor PRODUCTION OF ANTHOCYANIN PIGMENT1 (PAP1) (Fig. 4e) (Skaliter *et al*., 2019; Zvi *et al*., 2008). Similarly, the strategy taken in microbes of alleviating metabolic bottlenecks by harnessing the feedback‐insensitive enzymes AroG and PheA, bacterial DAHPS and CM/prephenate dehydratase, respectively, also yielded enhanced levels of vanillin in petunia (Fig. 4e; Oliva *et al*., 2015, 2017).

In all of the above studies in plants, a single genetic element was used. However, as shown in microbes, due to the complex nature of biochemical pathways leading to aroma compound production, to optimize carbon channelling, multiple genes need to be targeted. Thanks to advances in synthetic biology, multigene expression is now a routine practice in plants—it is no longer restricted to microbes. This strategy of using gene combinations proved successful in strawberry. Expression of petunia *isoeugenol synthase* (*IGS*) in strawberry fruit did not affect the concentration of either *t*‐anol or isoeugenol, which was nearly zero (Hoffmann *et al*., 2011). However, when in addition to *IGS* expression, flavonoid biosynthesis was blocked via suppression of the branchpoint enzyme CHS, a substantial increase in both compounds (ca. 400 ng/g FW) was achieved (Cna'ani *et al*., 2014; Hoffmann *et al*., 2011; Oliva *et al*., 2017; Zvi *et al*., 2008). Recently, Koeduka *et al*. (2021) also successfully implemented multigene expression for RK production in plants (Fig. 4f). Ectopic expression of BAS together with RZS1 in tobacco led to the accumulation of ca. 2 μg/g FW of RK and its glycoside. However, when metabolic flow in these BAS/RZS1‐expressing plants was enhanced via suppression of chalcone synthase (*CHS*), which competes with BAS for the substrate *p*CA, or by expression PAP1, a further three‐fold increase in RK production was obtained in the tobacco flowers, similar to levels in raspberry fruit (Koeduka *et al*., 2021). Coexpression of PAP1 with BAS/RZS1 enabled RK biosynthesis in the leaves as well, representing the plant's main biomass, probably through activation of phenylpropanoid pathway genes by PAP1 (Koeduka *et al*., 2021; Skaliter *et al*., 2019; Zvi *et al*., 2008).

**Figure 5:**
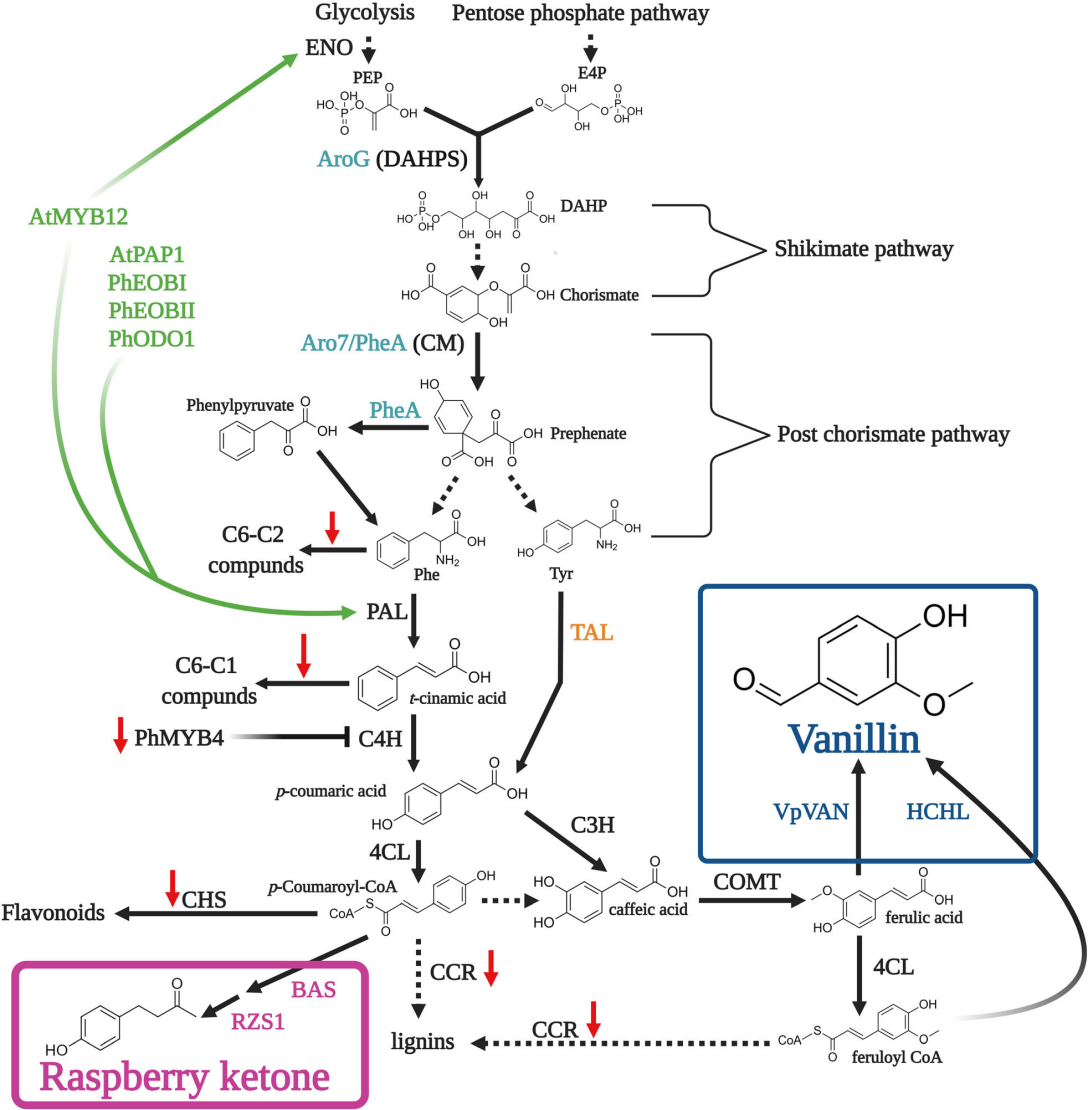
Proposed routes for enhancement of vanillin, raspberry ketone, and other phenylalanine (Phe)‐derived aroma compounds in plants. Alleviation of metabolic bottlenecks by feedback‐insensitive enzymes (cyan) in the shikimate (AroG) and post‐chorismate pathways (Aro7/PheA); upregulation of glycolysis and activation of PAL using transcription factors AtMYB12, PAP1, ODO1, and EOBI/II (green); use of TAL (orange) for utilization of tyrosine; knock out enzymes/repressors by CRISPR/RNAi (red arrows) at key branchpoints to ensure carbon flow directionality; and use of dedicated enzymes to complete the final biochemical steps towards vanillin (blue) or raspberry ketone (pink). Dashed arrows indicate multiple enzymatic steps. ENO, plastidial enolase.

The strategy of integrating numerous genetic elements, opening new routes and blocking unwanted fluxes (Fu *et al*., 2018) has yet to be used for vanillin engineering in plants. This strategy can be applied to suitable plant hosts, such as tobacco and pepper, and logically, to the vanilla orchid, once transformation and regeneration of this latter plant become available. The vanilla orchid is the most efficient natural producer of vanillin, despite the long pollination, growth and curing processes; and the recent publication of its genome (Hu et al., 2019) makes its use especially promising. Once it is possible to apply gene‐editing techniques, such as CRISPR to *Vanilla*, it will be possible to direct the flux towards phenylpropenes by, for example, knocking out negative regulators, such as the vanilla ortholog of *PhMYB4* and *CHS*, and enhancing total metabolic flux in the phenylpropanoid pathway by introducing feedback‐insensitive AroG and PheA with transcription factors, such as EOBI/II, ODO1, PAP1, and AtMYB12 (Fig. 5; Boersma *et al*., 2022; Fu *et al*., 2018; Oliva *et al*., 2015, 2017; Peled‐Zehavi *et al*., 2015; Spitzer‐Rimon *et al*., 2010, 2012; Zhang *et al*., 2015). However, overall, despite the potential of multigene combinations, they are not always straightforward (Tzin *et al*., 2015; Xie *et al*., 2016), and the crosstalk between different transgenes and its effect on metabolic flow should be carefully monitored and assessed.

## Future prospects

As detailed in this review, major progress has been achieved in delineating biochemical and molecular pathways yielding Phe‐derived compounds. In addition, we describe the numerous setbacks to efficient production of these compounds and present immediately applicable strategies to overcome them. Due to these molecules' multifaceted roles, that is, defence, signalling, attraction of pollinators, health benefits and contribution to food flavour, it is no surprise that so much effort has been invested in their production. At present, microbes, such as yeast, have the advantage because their products are regarded as GRAS. Moreover, aroma compounds, such as vanillin produced by genetically modified microbes are already commercially available (Altpeter *et al*., 2016). From an industrial standpoint, plants are lagging behind, as to our knowledge, no aroma compounds are commercially available from engineered plants. Here we review the next steps that should enable realizing the potential of molecular farming in these organisms.

There are various reasons for the current status—the main ones being technical and regulatory. From a technological/biological viewpoint, scientists have at their disposal a set of molecular tools that have yet to be exploited for molecular farming. For example, cellular localization of target genes and proteins is a crucial tool that enables utilizing several compartments together, with their respective intracellular pathways and substrates, as has been shown in both plants and microorganisms (Farhi *et al*., 2011; Kappers *et al*., 2005). Furthermore, transformation of organelles has many advantages, for example, plastid transformation may be more efficient than nuclear transformation due to the higher number of plastids in the cells, thereby potentially producing higher yields (Daniell *et al*., 2016; Maliga, 2004). Different combinations of *cis*/*trans*‐regulatory elements may further improve the expression of target genes and confinement of the product to specific tissues or compartments. In this respect, altering specific sites in the promoter regions of target genes allows fine‐tuning expression in cases of negative epistasis (Soyk *et al*., 2017). Inducible systems for gene activation may enable overcoming the many plant‐inherent problems that arise when dealing with metabolic fluxes that affect the host's well‐being, or with metabolites that are toxic to the host (Misra and Ganesan, 2021). To this end, an advanced chemical‐induced system that allows the initiation of cell‐specific production of specialized metabolites at predetermined times is being developed (Snir, 2020). Another major hurdle for metabolic engineering is that for many commercially important plants, including *Vanilla*, there are no efficient transformation/regeneration protocols (Altpeter *et al*., 2016). One way to overcome this is through viral‐based techniques, which bypass the need for tissue culture/regeneration, a major bottleneck in transgenesis as reported for numerous plants (Ellison *et al*., 2020; Honig *et al*., 2015; Hsu *et al*., 2015; Spitzer‐Rimon *et al*., 2012). A recent publication provides the tailwind for this technique, as the authors developed a transient viral‐based system to manipulate agronomic traits that can potentially be utilized to enhance aroma compounds in target plants (Torti *et al*., 2021). For many commercially important compounds, including vanillin, perhaps the biggest issue is that the pathways for its synthesis and metabolic fluxes have yet to be fully elucidated. Once the biochemistry is fully detailed, a host that can support production of the molecule of interest and is amenable to genetic manipulation/phytoviruses can be selected. Integration of the multiple metabolic engineering strategies discussed above with advanced agricultural techniques will allow maximizing the production efficiency of specialized metabolites for industrial purposes. Microalgae and synthetic plant cell‐based platforms serving as solar‐powered biofactories that combine the advantages of microbes and plants (Arya *et al*., 2020; Brey *et al*., 2020; Gimpel *et al*., 2015) may one day become a system of choice for metabolic engineering efforts.

Even if the technology can live up to its potential, regulatory limitations and public acceptance are still major setbacks. However, winds of change are blowing, one example being the approval of plant‐based vaccines, like the one for COVID‐19 developed by Medicago Inc. (Maharjan and Choe, 2021; Margolin *et al*., 2018). Furthermore, a gene‐edited tomato with enhanced levels of γ‐aminobutyric acid, which has attributed health benefits, is already commercially available in Japan (Nonaka *et al*., 2017; Waltz, 2022). The huge potential of molecular breeding can no longer be ignored, as is becoming clear to both legislators and consumers. However, the rise of plant platforms does not mean a dark future for microbes as producers of aroma compounds. As already noted, each system has its pros and cons. For example, microbes may be more suitable for wealthy countries with limited agricultural land, whereas plant molecular farming has an advantage in poorer regions, as plants generally have three basic needs: water, soil, and sun. Because some aroma compounds are worth more money than the crops, they may help local farmers. Furthermore, in the shadow of global anthropogenic‐related environmental changes, the ability to effectively produce molecules of interest while preserving plant–plant/pollinator interactions should significantly aid in sustaining the well‐being of both man and nature.

## Conflicts of interest

A.V. is an advisor to Pigmentum Ltd. Israel.

## Author contributions

O.S. and A.V. designed and conceptualized the review. All authors wrote the manuscript and approved it.
